# Nervous Persons

**Published:** 1871-11

**Authors:** 


### NERVOUS PERSONS

Would often be very greatly benefited, if they would put on their hats or bonnets, when they find themselves in a restless condition, and visit some friend, a mile or more away ; perchance that same friend may be in the self-same condition, but the very meeting of friends, the exhilaration of inquiries and answers, and passing comments, often wake up to newness of life; and the very heart that was a short time before almost weighed to the earth, bounds upwards and is filled with joyousness.

Be assured we are losing much of the social enjoyments of fifty years ago. It requires so much of our time nowadays to keep our houses in order, to sweep, and dust, and arrange, that by the time a tidy housekeeper has got through with her morning's work, it is so late in the day, or she is so tired, that there is really not the strength to go out and take a walk, or make a friendly call, let alone spend a whole evening. Thus it is that an advanced civilization tends to make us the greater slaves of form and ceremony and fashion. One of the tidiest housekeepers in a very large city was taken suddenly ill; and as she laid in her beautiful chamber and cast her eyes over the costly furniture, she exclaimed, in tones O how mournful, "I have lived a useless life!" and died. It was true; a very large part of her whole existence had been spent in dusting furniture. He who is so busy in keeping the house in order, that no time is left for practicing the sociabilities of life, is robbing himself of very much that tends to make that life happy.

